# Vavilov's Collection of Worldwide Crop Genetic Resources in the 21st Century

**DOI:** 10.1089/bio.2018.0045

**Published:** 2018-10-12

**Authors:** N.I. Dzyubenko

**Affiliations:** The N.I. Vavilov All Russian Institute of Plant Genetic Resources, Federal Research Center, St. Petersburg, Russia.

**Keywords:** biodiversity, breeding, conservation, food security, genetic collection, plant genetic resources, seeds, Vavilov

## Abstract

N.I. Vavilov was among the first scientists who recognized the high potential value of plant genetic resources (PGR) for humankind. In addition to his fundamental work on the centers of crop origin, he emphasized the importance of collection and *ex situ* conservation of cultivated plants and their wild relatives, to make them available for breeding programs and for future generations. Vavilov's ideas formed a solid scientific basis for the long-term efforts on securing PGR in *ex situ* genebanks, both internationally and in Russia. The collection of seeds and living plants at the N.I. Vavilov All Russian Institute of Plant Genetic Resources (VIR) is one of the oldest in the world. The size of the collection increased from 301 accessions in 1901 to over 330,000 accessions in 2017, now representing 64 botanical families, 376 genera, and 2169 species. Acquisition was mainly focused on crops that are suitable for cultivation in Russia such as potatoes, barley, wheat, sorghum, beans, vegetables, forage species, and many others. For over a century, VIR has been providing the materials for breeding programs and research, which resulted in developing new cultivars with unique characteristics such as high yield combined with deceased resistance, improved storability, cold and drought tolerance, or ability to grow on deserts and polluted lands. The main field collection near St. Petersburg and 11 main branches across the country covering a wide spectrum of climatic conditions combined with modern seed storage, *in vitro* and cryobank facilities, and molecular laboratories form a solid platform for breeding, regeneration, and evaluation of accessions in the collection. This article gives a brief overview of VIR as the leading genebank and breeding center in Russia, its main activities in conservation and utilization of PGR for national food security and its role in developing national policies in this area.

## Plant Genetic Resources and the History of N.I. Vavilov All Russian Institute of Plant Genetic Resources Collection

*E**x situ* collections of cultivated plants and their wild relatives are the basic components of food and environmental security of each sovereign country, including Russia. Their relevance and strategic importance have been recognized in the past decades due to enormous genetic erosion and worldwide extinction of crop varieties, species, and even genera. According to the Food and Agricultural Organization of the United Nations (FAO) (1998), 75% of the world's crop genetic diversity was lost in the 20th century. Moreover, the number and assortment of cultivated plants and their wild species have been significantly reduced. Only 30 agricultural crops provide for 95% of humankind's requirements in high-calorie plant-derived food, and 4 of them, rice, wheat, maize, and potatoes, cover more than 60% of our demand in energy and proteins.

Contemporary losses of the diversity and a small number of crops used in production under the global and local climate changes present a serious threat to the entire worldwide community and aggravate the problem of food security. Therefore, in Russia as in many other countries, conservation and sustainable utilization of PGR have acquired national and strategic importance.

The Russian collection of plant genetic resources (PGR) *ex situ* is one of the oldest in the world. It was established in October 1894 as the Bureau of Applied Botany under the Scientific Committee at the Ministry of State Property of the Russian Empire. The Bureau gradually evolved into the N.I. Vavilov Institute of Plant Genetic Resources, or VIR, which became the national center of conservation, research, and utilization of PGR.

The present status of VIR's collection is the result of the collective efforts of several generations of world-renowned scientists and explorers. The central, and probably the most internationally acknowledged figure in VIR history is Nikolai Ivanovich Vavilov. He was among the first scientists who recognized the exceptional importance and potential value of collecting and preserving the genetic diversity of crop varieties and their wild relatives for humankind. He also laid the foundation for the national and global strategies in PGR conservation and sustainable utilization. Vavilov's theory of the centers of crop origin, his law of homologous series in hereditary variation, fundamental works on geographic distribution of genes, the role of source material in breeding practice, and other publications are internationally renowned. Science historians and Vavilov's biographers usually highlight two stages in his program development. The first stage (1917–1929) was mainly devoted to the collection and research of world genetic resources of staple crops and their wild relatives. The second stage (1929–1940) was mainly focused on a wide-scale scientific synthesis of the acquired knowledge and development of a theoretical basis of crop biology and breeding.^1,2^

**FIG. 1.**
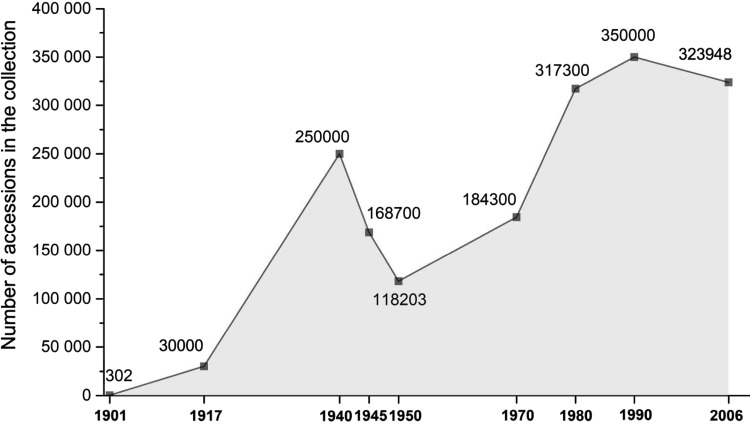
Dynamic change of the number of accessions in VIR collections in 1901–2006. VIR, N.I. Vavilov All Russian Institute of Plant Genetic Resources.

The active research and collection work commenced by Vavilov and his team was continued throughout 20th century, but only hampered by World Wars I and II. By 1901, VIR collection of PGR was composed of 301 accessions. In 2015, this number increased to 325,000 unique accessions (Fig. 1). During the past 90 years, VIR conducted over 1558 collection expeditions in the former USSR regions and 282 foreign countries. Priority was given to crops and accessions that were potentially suitable for cultivation under the large spectrum of Russian climatic conditions varying from subtropical southern parts to permafrost regions in the north, from plains to mountain areas, coastal, and dry lands. This climatic range justified high diversity among accessions in the collections, which is currently considered one of the richest in the world, based on the diversity of represented alleles and traits.^3–5^

## Current Status of the Collection

The activities in monitoring, collecting, conservation, documentation, comprehensive study, and effective utilization of global PGR are ongoing and creatively developed on the basis of modern science and technology achievements, with due regard to new economic and political trends.

The dynamic change of accession numbers in VIR collection during the past years is presented in Table 1. As of 2017, the VIR collection holds 346,666 accessions of PGR and crop wild relatives representing 64 botanical families, 376 genera, and 2169 species. This includes vegetatively propagated perennial crops maintained in the field genebanks (29,611 accessions), *in vitro* (750 accessions), and in cryopreservation (1824 accessions). Being the fourth in the world in the number of the preserved accessions, VIR maintains probably the richest botanical, genetic, geographic, and ecological diversity of plant germplasm.

**Table 1.** Recent Dynamics of the Accession Numbers in VIR Collection Classified by Staple Crop Groups

	Number of accessions^*^					
*Crop groups*	*2011*	*2012*	*2013*	*2014*	*2015*	*2016*
Wheat, triticale, aegilops	51,662	50,568	51,234	51,580	52,409	52,658
Rye, oat, barley	36,509	36,841	36,885	36,549	36,581	36,688
Small-grain groat crops	48,358	48,309	48,606	48,529	48,529	48,529
Perennial forage crops	30,489	30,963	31,311	31,366	31,366	31,366
Grain legumes	45,845	46,141	45,438	46,317	46,135	46,344
Oil and fiber crops	27,471	27,517	27,680	27,970	27,970	28,119
Potato	9647	9239	8864	8692	8958	8570
Vegetables	50,138	50,205	50,088	49,971	50,019	50,089
Fruits and berries	23,734	23,558	23,073	22,750	22,750	22,750
Total	323,853	323,341	323,179	323,724	324,717	325,113

^*^ The decrease in number of accessions in the collection observed for some crop groups for several years reflects the extensive work on elimination of duplicates.

VIR, N.I. Vavilov All Russian Institute of Plant Genetic Resources.

**FIG. 2.**
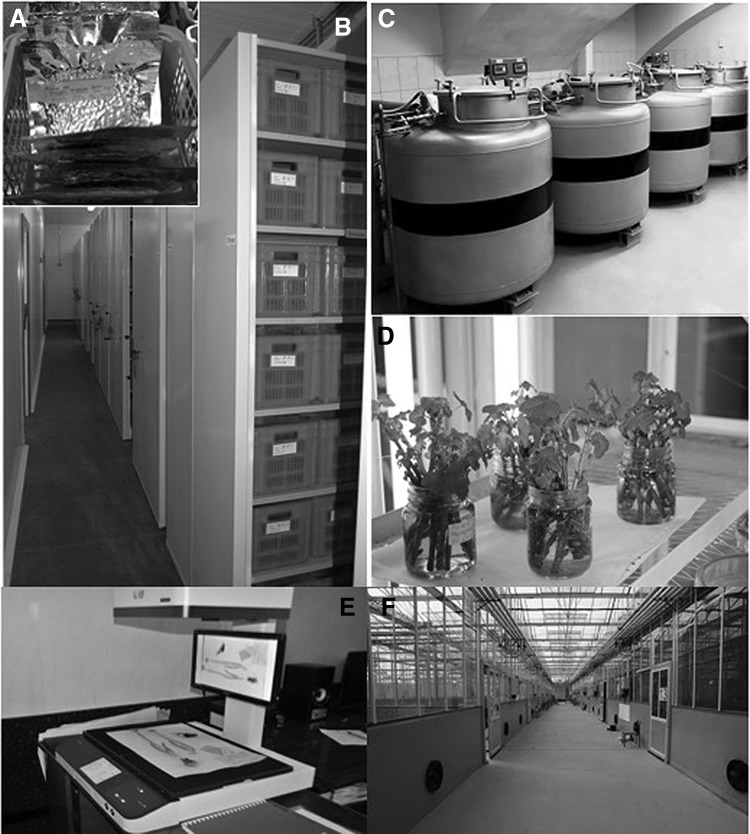
Main facilities of VIR genebank in St. Petersburg, Russia. **(A)** Storage of dried seeds in vacuum-sealed aluminum packs; **(B)** long-term seed storage at −10°C equipped with moving shelves; **(C)** VIR cryobank; **(D)** regrowth of *Ribes* scions after cryogenic storage; **(E)** digitalizing VIR herbarium; **(F)** “Phitotron” greenhouse complex for growing plants under the controlled environment opened in 2015.

**FIG. 3.**
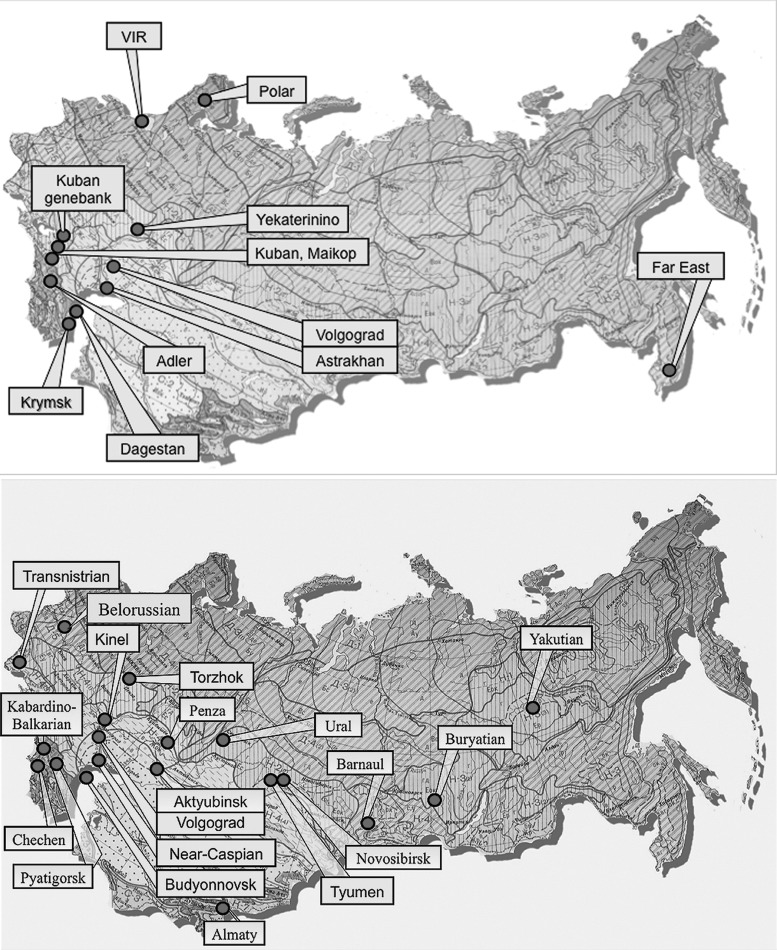
Schematic map of VIR regional branches (*top*) and experimental stations (*bottom*) as of January 2017.

Every year the genebank is replenished with 1000–3000 new accessions that are securely preserved as seeds, vegetative and generative organs, test-tube plants in tissue culture or DNA in controlled environments of low-temperature, and *in vitro* or cryogenic storage (Fig. 2). The main facility for seed conservation in St.Petersburg has five cold rooms for seed storage at −10°C and 4°C, *in vitro* and phytosanitary laboratories, and a cryobank and herbarium operated according to FAO genebank standards (Table 2 and Fig. 2). In addition to the main facility, VIR operates 11 main branches and experimental stations distributed all over the country to cover most of the climatic zones (Fig. 3). Table 3 illustrates the significant contribution of VIR regional branches and stations to the main activities of multiplication, regeneration, and conservation of PGR. In addition, in 2017, 4885 species of crop wild relatives have been identified and screened for their potential introduction into conservation *in situ* and in a genebank. Seed-producing accessions from the main collection in St. Petersburg are safety duplicated at the Kuban seed genebank.

**Table 2.** Low-Temperature Storage of Seed Accessions at VIR Central Seed Storage Facilities in St. Petersburg (as of January 1, 2017)

		*Active collections*	
*Crop group*	*Base collection−10°C*	*−10°C*	*4°C*
Wheat, aegilops, and triticale	9430	7515	14,910
Oat, rye, and barley	14,025	9199	16,008
Groat crops	8359	8200	27,766
Grain legume crops	9387	15,364	33,539
Vegetable and cucurbit crops	11,915	11,191	19,312
Oil and fiber crops	8602	17,025	14,707
Perennial forage crops	8434	18,941	5810
Potato	834	2314	—
Fruit crops	221	420	—
Laboratory long-term storage	864	—	—
Total	114,125	90,169	132,052

**Table 3.** Conservation, Multiplication, and Regeneration of VIR Collections in 2017

	*Number of accessions*	
*Activity*	*Main station*	*Regional stations and branches*
Conservation (field genebanks)	13,375	20,167
Regeneration due to decreased viability	12,306	28,688
Multiplication, including for introduction into the long-term storage	2992	14,253

At present, genebanks have an opportunity to use molecular markers to monitor the global processes steering the dynamics of plant diversity involved in breeding practice. According to the researchers, the greatest loss of genetic diversity accompanies the domestication phase of a crop, so insufficient representation of the crop's wild relatives in the collections is very likely to invoke disappearance of valuable alleles that were not of direct interest to our remote predecessors, but are quite vital at the present moment. For many years, VIR has been working toward collecting and evaluating of crop wild relatives and their involvement in breeding programs. According to recent data, over 1680 species of crop wild relatives occur in Russia as native populations representing ∼62 families; the largest of those are Poaceae, Fabaceae, Rosaceae and Alliaceae. These species have been targeted in recent VIR collection missions. In 2017, for example, 26 collecting missions were successfully launched over the territory of the former Soviet Union.

The VIR herbarium hosts 376,825 specimens that are used as a reference collection. VIR currently runs an ambitious program to create a Virtual herbarium. With the target rate of digitalizing 1900 herbarium specimens per month, about 110,000 images should be created and uploaded to the electronic database by 2021 (Fig. 1). In addition, efforts have been made to digitalize the most precious and significant part of VIR library with a target to have 210,000 books in electronic form by 2021.

## The Use of VIR Collection for Improvement of PGR and Agricultural System

It is hard to overestimate the impact of the VIR collections on breeding, food production, and development of environmentally safe agriculture in Russia and former Soviet Union countries. Suffice it to say that for the past six decades, the yield of cereal crops increased two to five times as a result of implementing unique cultivars developed by Russian and former USSR breeders based on the accessions from VIR's genebank. For example, introduction of potato wild species collected during expeditions in South and Central Americas into breeding practice allowed the widespread cultivation of this crop over all of Russia due to acquired traits of earliness, improved storability, and resistance to diseases and pests. As a result, by the end of the 20th century, potato yield increased two to four times. Using the traits of earliness and cold tolerance in the breeding of wheat, oat, sunflower, soybean, cotton, rice, maize, and other crops helped to extend their cultivation areas far into the north. Now, for example, maize is grown for grain almost 2000 km further north and 10,000 km further east than 50 years ago.

Recent data on distribution of VIR materials to breeding centers and genebanks in Russia and internationally are presented in Table 4.

**Table 4.** Distribution of Materials from VIR Collection in 2014–2016

*Institution (type of distributed material)*	*2014*	*2015*	*2016*
Distribution within Russia			
Breeding centers (new accessions)	2813	446	745
Breeding centers (breeding materials)	1200	1991	1856
Breeding centers (prebreeding materials)	31	89	85
Breeding centers (targeted collections, or minicores)	1963	2112	2351
Other research institutions (all materials)	3785	2380	2533
Northwest regions^*^	1241	702	2023
Total in Russia	11,033	7720	9593
Genebanks and research institutions outside Russia	2692	2797	1908
Total	13,725	10,517	11,501

^*^ Distribution in the frame of the long-term governmental program to support the regional centers and farmers in the Northwest areas of the country. Due to program requirements, distributions to these regions were recorded separately.

Eighty percent of all varieties and hybrids currently cultivated in the country have VIR's accessions in their pedigree. For example, over 300 rye, oat, and barley cultivars were released on the basis of VIR materials, and 120 of them have already been used in agricultural production. Identification of a dominant dwarf gene in VIR rye collection initiated the world's general trend in winter rye breeding—development of nonlodging cultivars. Short-stemmed cultivars bred using this gene now occupy ∼80% of rye cropping areas in the Russian Federation, and their economic value amounts to billions of rubles annually.

VIR collections and research significantly contributed to the introduction of new food species into cultivation in Russia, such as spelt wheat, amaranth, Chinese cabbage, *Stevia*, cherry plum, sea buckthorn, *Actinidia*, honeysuckle, and so on, and to growing crops on acidic, desert, and polluted lands, including phytoremediants, ameliorants, and edificators. For example, new arid forage crops have been introduced into the near-Caspian areas, such as *Eurotia*, *Kochia prostrata*, and saxaul.

The collection plays the leading role in conservation and improvement of major crops and mitigating their vulnerability to biotic and abiotic stressors. Out of nearly 330,000 accessions held at VIR's genebank, ∼30% are already extinct in nature or lost by crop producers. These accessions often carry genes valuable for breeding and crop production, such as genes of resistance to various pathogens; hence, they may be in demand at a certain stage. For example, using the genetic diversity of wild potato species lost in natural environments, but preserved in the collections, allowed potato breeders to develop cultivars resistant to late blight and other diseases. Representatives of remote and rare species, crop wild relatives, landraces, and varieties with diverse pedigrees, when used in breeding practice, expand the pool of valuable genes determining agronomic and economic traits, broaden the hereditary base of the developed cultivars, reduce their vulnerability, and help them resist stressors.^6–10^

Using accessions from VIR collection allowed the revival of breeding and cultivation of crops previously discarded from the production. These included industrial crops such as false flax, *Taraxacum kok-saghyz*, oil-bearing spurge, castor bean, *Crambe*, or *Eruca*, which attracted keen attention when searching for new raw materials, alternative fuels, natural rubber or nonfreezing lubricants.

Comprehensive studies during the past 10 years allowed VIR's researchers to identify and develop over 20,000 sources and 500 donors of gene and polygene alleles valuable for breeders, and establish numerous trait-specific and genetic collections (mini-cores) of major crops for targeted utilization in breeding programs. New effective alleles, rare ones or those earlier ignored by breeders, which determine photoperiodic sensitivity and response to vernalization, resistance to pathogens and other traits, were identified and mapped. Marking and molecular mapping quantitative trait loci allowed researchers to find, identify, and perform a controlled transfer of chromosome loci determining expression of economically useful traits such as resistance to biotic and abiotic stressors, quality, and productivity. Genetic diversity of accessions in the collection studied by conventional technologies and innovative molecular genetic approaches, including genomics, molecular marking, mapping, and bioinformatics, serves as a powerful base of valuable source material and an instrumental mechanism of effective breeding development. We believe that the diversity of the collection materials, provided that they are safely conserved and sustainably utilized, may promote breeding technologies and priority trends in the 21st century breeding, which are oriented toward the development of high-quality food, optimization of animal feed production, global climate change, “nordification” of crop production, development of novel agro-, bio-, food, chemical, and industrial technologies, biologization and ecologization of agriculture, and resource- and energy-saving policies.^11,12^

## Vavilov Institute and National Strategy for Genetic Resources Conservation and Food Security

As the national genetic resources center with over 120 years of history, VIR has been responsible for mobilization, conservation, evaluation, and utilization of PGR in Russia and involved in the development of relevant governmental programs and legal instruments. In addition to VIR collection, ∼50,000 accessions of PGR are held by other organizations in Russia: research institutions, breeding centers, universities, botanical gardens, commercial plant breeding and seed production companies, and on farms. Over 1600 species of crop wild relatives occur in Russia within their natural populations—they are potential carriers of alleles valuable for breeding. Many of them are now under the threat of extinction and require measures toward their safeguarding.^1,13^

The problems of PGR conservation in genebanks, within natural plant communities and on farms, and their sustainable utilization are listed among the five top priorities of national security in most of the world's countries, including Russia. These problems need to be solved both at national and international levels, because countries are not self-sufficient and depend on each other in the context of PGR. To find a successful solution to such problems, relevant international documents have been developed such as the Convention on Biological Diversity, 1992; Global Plan of Action for Plant Genetic Resources for Food and Agriculture, 1996, 2011; International Treaty on Plant Genetic Resources for Food and Agriculture, 2004; and Nagoya Protocol on Access to Genetic Resources, 2011. However, with new challenges of globalization and emergence of new technologies, climate change and rapid genetic erosion, profound modifications should be made in the national and international policies in the area of crop agricultural biodiversity conservation and utilization.

The importance of VIR's collections and activities in providing solutions to plant breeding and production problems in Russia as well as their role in solving the problem of food supply has been emphasized at several international forums. The former minister of agriculture, E.B. Skrynnik, informed the delegates at the FAO World Summit on Food Security (Rome, 2009) that Russia is ready to make VIR's collection available for the worldwide community, considering possible climate changes and the common interest of all countries in the reduction of the starvation and poverty level in the world. However, Russia has not yet joined the International Treaty on Plant Genetic Resources for Food and Agriculture; hence, international distribution of accessions from VIR genebank has been accomplished based on bilateral agreements rather than SMTA.

In 2006, VIR developed and proposed the draft of the National Program “Conservation and Sustainable Utilization of Crop Genetic Resources,” which envisaged necessary budget funding for effective coordination of PGR activities at the national, regional, and international levels. Despite the first approval, the program was not funded or implemented. Later, genetic resources conservation was prioritized in the list of national programs, which was approved by the President and Government of the Russian Federation. Most recently, joint efforts of the Russian Ministry of Agriculture and VIR helped to develop and coordinate at the ministerial level of stakeholders the draft Federal Law “Concerning Plant Genetic Resources for Agricultural Production,” which will regulate the activities in the areas of collection, regeneration, storage, research, and sustainable utilization of PGR and their collection for agricultural production in the Russian Federation.

The main idea of the proposed National Program is to combine governmental and nongovernmental measures to create the most favorable environment for secure *ex situ* and *in situ* conservation of genetic resources, development of fundamental and applied research in the area of agricultural biodiversity, avoiding duplication of activities, enhancement of valuable plant diversity mobilization possibilities, expansion of the national genetic diversity by targeted collecting missions over Russia and other countries, as well as through international germplasm exchange, and efficient and sustainable utilization of bioresources with the help of new technologies and scientific achievements.

The National Program will support effective coordination of PGR-related activities within the country and help to establish solid partnerships between governmental and private institutions holding *ex situ* collections. It will also open the floor for further discussion and adoption of new international agreements, including the issues of access and benefit sharing as well as to implement large-scale and mutually beneficial international and regional cooperation, while observing the national interests.

Thus, the proposed National Program on Genetic Resources of Cultivated Plants and Their Wild Relatives of the Russian Federation represents the basic instrument and main mechanism in achieving the goals of mobilization, conservation, and sustainable utilization of agricultural biodiversity components, as it would help to implement relevant activities at the national and international levels. The National Program will become an integral part of the National Strategy and National Plan of Action in the area of biodiversity conservation in the Russian Federation.
